# Evaluating the bone‐regenerative role of the decellularized porcine bone xenograft in a canine extraction socket model

**DOI:** 10.1002/cre2.361

**Published:** 2020-12-01

**Authors:** Yuan‐Wu Chen, Meng‐Yen Chen, Dar‐Jen Hsieh, Srinivasan Periasamy, Ko‐Chung Yen, Chao‐Tang Chuang, Hung‐Chou Wang, Fan‐Wei Tseng, Jer‐Cheng Kuo, Hua‐Hong Chien

**Affiliations:** ^1^ Division of Oral and Maxillofacial Surgery Tri‐Service General Hospital Taipei Taiwan; ^2^ School of Dentistry National Defense Medical Center Taipei Taiwan; ^3^ Division of Oral and Maxillofacial Surgery, Department of Stomatology National Cheng Kung University Hospital Tainan Taiwan; ^4^ R&D Center ACRO Biomedical Co., Ltd. Kaohsiung Taiwan; ^5^ Division of Periodontology, College of Dentistry Ohio State University Columbus Ohio USA

**Keywords:** bone regeneration, carbon dioxide, chromatography, supercritical fluid, heterograft

## Abstract

**Objective:**

To evaluate the efficacy of a novel decellularized porcine bone xenograft, produced by supercritical carbon dioxide extraction technology, on alveolar socket healing after tooth extraction compared to a commercially available deproteinized bovine bone (Bio‐Oss®).

**Materials and methods:**

Nine dogs (about 18 months old and weighing between 20 kg and 30 kg) underwent extractions of lower second to fourth premolars, bilaterally. The dogs were randomly selected and allocated to the following groups: Group 1: control unfilled socket; Group 2: socket filled with decellularized porcine bone xenograft (ABCcolla®) and covered by a commercially available porcine collagen membrane (Bio‐Gide®); Group 3: socket filled with Bio‐Oss® and covered by Bio‐Gide® membrane. One dogs from each group was sacrificed at 4‐, 12‐, and 24‐week to evaluate the socket healing after tooth extraction. The mandible bone blocks were processed without decalcification and specimens were embedded in methyl methacrylate and subjected to histopathology analyses to evaluate the bone regeneration in the extraction sockets.

**Results:**

At 24‐week after socket healing, ABCcolla® treated defects demonstrated significantly higher histopathology score in new bone formation and bone bridging, but significantly lower score in fluorescent labeling than those of the Bio‐Oss®. In the microphotographic examination, decellularized porcine bone xenograft showed similar characteristics of new bone formation to that of Bio‐Oss®. However, there was significantly less remnant implant materials in the decellularized porcine bone xenograft compared to the Bio‐Oss® group at 24‐week. Thus, the decellularized porcine bone graft seems to have promising bone regeneration properties similar to that of Bio‐Oss® with less remnant grafted material in a canine tooth extraction socket model.

**Conclusions:**

Within the limits of the study, we concluded that ABCcolla® treated defects demonstrated significantly more new bone formation and better bone bridging, but less amount of fluorescent labeling than those of the Bio‐Oss® group. However, clinical studies in humans are recommended to confirm these findings.

## 1 INTRODUCTION

The key processes of healing in an extraction socket have been well documented in both dogs and humans (Amler, 1969; M. G. Araujo & Lindhe, 2005; Cardaropoli et al., 2005; Pietrokovski & Massler, 1967; Trombelli et al., 2008), although bone remodeling has been reported to be three to five times quicker in dogs than in humans. The healing process of an extraction socket can be divided into three sequential phases including inflammatory, proliferative and modeling/remodeling (M. G. Araujo et al., 2015). In general, following tooth extraction, most of the socket is filled with a blood clot during the first 3 days of healing. Ultimately the blood clot is replaced by granulation tissue comprised of inflammatory cells, vascular sprouts, and immature fibroblasts. The granulation tissue is steadily replaced by a temporary connective tissue matrix containing immature collagen fibers and cells. Osteoid formation begins as early as 1 week after tissue remodeling. Immature woven bone can be identified as early as 2 weeks after tooth extraction and remains in the socket for several weeks. As bone remodeling and modeling progress, the woven bone is replaced with lamellar bone or bone marrow and the socket walls undergo bone resorption. Consequently, bone remodeling results in the reduction of both alveolar ridge width and height.

The extent and magnitude of ridge resorption post‐extraction typically result in a certain degree of bone loss in both horizontal and vertical directions. It has been demonstrated that loss of alveolar ridge height and width is an irreversible phenomenon after tooth extraction (M. G. Araujo & Lindhe, 2005, 2009; Schropp et al., 2003). A systematic review investigating the magnitude of human ridge dimensional changes following tooth extraction has revealed that a horizontal bone loss of 29%–63% and a vertical bone loss of 11%–22% occur 6 months after tooth extraction (Tan et al., 2012). It is expected that half of the original alveolar ridge width will be lost in 6 months after tooth extraction. Furthermore, extensive resorption of the alveolar ridge may have a significant impact on implant‐supported therapy (Seibert & Salama, 1996). Therefore, alveolar ridge preservation (also known as socket preservation) after tooth extraction often leads to a decrease in post‐extraction dimensional loss. Therefore, alveolar ridge preservation is an effective therapy to prevent or minimize dimensional bone loss after tooth extraction (G. Avila‐Ortiz, Chambrone, et al., 2019; G. Avila‐Ortiz, Elangovan, et al., 2014; Bassir et al., 2018).

Xenogeneic bone replacement materials are obtained from non‐human species and have been widely used in clinical periodontal regeneration (Sheikh et al., 2017). Processing of the xenogeneic bone tissue must be conducted to warrant the elimination of immunogenic components and pathogens before its clinical use. Different methods have been applied in the processing of non‐human tissue, and innovative supercritical carbon dioxide (SCCO_2_) technology is one of those methods. The regenerative property of the porcine grafting material can be improved by a decellularization process using SCCO_2_ extraction technology (Huang et al., 2017). Using CO₂ as a solvent in the SCCO₂ technique has many advantages. CO₂ is natural, safe, non‐toxic, non‐corrosive, non‐flammable, easily accessible, and cost effective (Fages et al., 1994). There is no solvent residue or off‐odors, and supercritical CO₂ easily solubilizes hydrocarbons including oil and lipids for their removal. However, polar molecules such as amino acids and proteins are preserved. The use of SCCO_2_ in sterilizing amniotic membrane‐derived tissue grafts while simultaneously preserving their biological characteristics has been well‐documented (Wehmeyer et al., 2015). Furthermore, SCCO_2_ has been used for bone delipidating, because it possesses a good solvent capacity for lipids (Fages et al., 1994). In particular, the mechanical properties of bone can be enhanced due to cross‐linking of collagen chains. A significant reduction in the antigenicity by SCCO_2_ without compromising the strength of bone, similar to native bone, has been reported (You et al., 2018).

With this theoretic background, it was hypothesized that the decellularized porcine bone xenograft, created by SCCO_2_ extraction technology, exhibits comparable outcome to that of a commercial deproteinized bovine bone (Bio‐Oss®) in socket healing of an extracted tooth in dog mandible. The purpose of this study was to evaluate and compare bone regeneration in canine dental sockets when using decellularized porcine bone xenograft or Bio‐Oss®.

## 2 MATERIALS AND METHODS

### 2.1 Preparation of decellularized porcine bone (ABCcolla® dental bone graft)

Porcine bones were purchased from Tissue Source, LLC (Indiana, USA). The bone was cleaned of residual tissues and washed with phosphate‐buffered saline (PBS). The porcine femur was prepared by first sectioning into circular discs using a mechanical saw, followed by cleaning in an ultrasonic bath. Then, the cleaned circular bone sections were ground into granules (0.25–1 mm) using a mechanical grinder before undergoing the SCCO_2_ decelluarization process. The bone was placed on a tissue holder, which was then inserted into a SCCO_2_ vessel system (Helix SFE Version R3U, Applied Separations Inc., Allentown, PA) containing 30 ml 60% ethanol. The SCCO_2_ system was then operated at 350 bar and 45°C for 80 min to produce decellularized porcine bone (ABCcolla®, ACRO Biomedical Co. Ltd., Kaohsiung, Taiwan). FDA approved and commercially available Bio‐Oss® and Bio‐Gide® (porcine collagen membrane) were purchased from Geistlich Pharma North America Inc. (Princeton, NJ).

### 2.2 Study animals

All the experimental procedures used in this study were performed in accordance with the National Institutes of Health Guide for the Care and Use of Laboratory Animals and were reviewed and approved by the PharmaLegacy Laboratories IACUC (PharmaLegacy Laboratories, Shanghai, China *is fully accredited* by the Association for Assessment and *Accreditation* of Laboratory Animal Care International). A total of nine skeletally mature Labrador retriever dogs (about 18 months old and weighing between 20 kg and 30 kg) bred exclusively for biomedical research purposes were obtained from Jiagan Biological Technology Co., LTD (Shanghai, China). Pre‐ and post‐operatively, the animals were kept in kennel cages, received appropriate veterinary care with free access to water and standard laboratory nutritional support throughout the study period.

### 2.3 Surgical procedures

Extraction of mandibular premolars was performed under local and general anaesthesia. Surgical extraction of mandibular premolars (P2 to P4) was performed under general anaesthesia with Atropine (0.4 mg/10 kg body weight, i.m.) and Zoletil (10 mg/kg, i.m.) 30 min before surgery. The dogs were placed on a heating pad, intubated and inhaled with 1%–3% isoflurane and monitored during the surgery. After disinfection of the surgical site with 10% povidone‐iodine solution/1% titratable iodine, 2% lidocaine HCl with epinephrine 1:100,000 was administered by local infiltration at the buccal and lingual aspects. An intra‐sulcular incision was made around the neck of all four premolars with two vertical releasing incisions placed on buccal aspect extending beyond the muco‐ginigval junction. Full‐thickness mucoperiosteal flaps were elevated on both buccal and lingual aspects to expose the crestal bone. The three premolars (P2 to P4) on each side of the mandible were hemisected vertically with a dental carbide bur to separate all teeth into single root segments. Extraction was then carried out carefully with the use of forceps and elevators without damaging the alveolar cortical bone. All extraction sockets were checked visually after irrigation with 0.9% saline solution.

Nine dogs were randomly divided into three groups: Group 1: control (unfilled); Group 2: the socket was filled with ABCcolla® dental bone graft and covered by Bio‐Gide® membrane; Group 3: the socket was filled with Bio‐Oss® and covered by Bio‐Gide® membrane. The flaps were released by periosteal incision in order to mobilize the flap to achieve a tension‐free primary wound closure. The soft tissues were closed and sutured with absorbable polyglactin 910 (Vicryl 4–0, Ethicon™; Johnson & Johnson Medical Devices Companies, New Brunswick, NJ), in which the post‐extraction sockets were completely covered with the mobilized flaps for the control group, and the Bio‐Gide® membranes were mainly covered by the flaps for the other two groups. Hydration was maintained by intravenous infusion with 0.9% NaCl solution during the whole surgical procedure. Postoperative pain management and wound care were provided until the animals were returned to activities ad libitum. Softened dog food was provided during the first‐week post‐surgery and wound healing was monitored closely to avoid infection.

### 2.4 Postoperative care

Animals were observed throughout the postoperative period and received the analgesic buprenorphine (0.02 mg/kg, i.m.) for two additional doses at about 8 hours apart for 3 days. Antibiotic injection of Ceftriaxone (50 mg/kg, i.m.) was given at end of surgery and twice a day thereafter for 3 days.

### 2.5 Animals sacrifice

Tetracycline‐calcein double‐labeling was used in this study to assess the new bone growth rate. All animals received calcein (10 mg/kg, s.c.) and Tetracycline (10 mg/kg, i.v.) injection once at 2 and 9 days, respectively, prior to sacrifice, to be able to visualize new mineralizing tissue under fluorescent microscopy. The animals were humanely euthanized by an overdose of pentobarbital (100 mg/kg) at the end of 4‐, 12‐, and 24‐week (*n* = 3, with three per group and one at each time point per group) after the surgery. At necropsy, the wound healing was examined and photographed. Dog mandibles were microradiographed in a digital X‐Ray machine (Faxitron model MX20; Faxitron Bioptics, LLC., Tucson, AZ) to document the defect locations and orientations.

### 2.6 Biomechanical analysis

The healed extraction socket from both sides of the mandible was sectioned to generate two rectangular bone blocks. Each bone block contained the healed extraction socket of P2, P3, and P4 region. The biomechanical testing was performed using an MTS testing machine (MTS 858 Mini Bionix® II Biomaterials Testing System, Eden Prairie, MN). Only bone blocks collected from dogs euthanized at 24‐week were tested for the compression analysis. The specimen was placed on a specially designed platform with a self‐aligning function to ensure vertical compression. The sampling frequency was set at 500 Hz with a probe diameter of 2.0 mm. A pre‐load of 40 N with 30 s of accommodation time followed by a continuous and progressive load at a rate of 2 mm/min was applied. The first peak force detected during the test was recorded as the ultimate strength. The displacement versus force was recorded to calculate the maximal load. Maximal load in Newtons, stiffness in Newtons/mm, and energy to the maximal load in millijoules were measured and recorded in the biomechanical analysis of the bone blocks.

### 2.7 Histological preparation and histopathological analysis

All samples were immersed in 10% neutral buffered formalin for histological preparation, followed by dehydration with gradient ethanol solution using an automatic tissue processor (Shandon Excelsior ES™, Thermo Fisher Scientific Inc., Waltham, MA, USA) and finally embedded in methyl methacrylate. Undecalcified hard tissue slicing (100 μm) were then made using a cutting and grinding system (EXAKT 400CS; EXAKT Technologies, Inc., Okalahoma City, OK) and stained with Masson's trichrome for semi‐quantitative assessments of hard tissue healing, including the new bone fill. Masson's trichrome staining was processed following the manufacturer's instruction provided in the kit (HT15‐1KT; MilliporeSigma Inc., St. Louis, MO). Unstained sections were observed for tetracycline‐calcein labels under fluorescence microscopy for the measurement of bone formation. Histologic images were photographed with a camera (Nikon Optiphot‐2; Nikon Instruments Inc., Melville, NY) and subjected to an ImageJ software for analysis.

Histopathologic scoring is a tool by which semi‐quantitative analysis of bone healing in tissue‐engineer‐treated defects can be achieved (Han et al., 2018). A histological scoring system (Table 1), adapted from Zubery et al. (2007) with modifications created by Dr Mei‐Shu Shih, a pathologist from PharmaLegacy Laboratories, Shanghai, China, was used for the evaluation of bone regeneration. This histological scoring system has been used in PharmaLegacy Laboratories to quantitatively evaluate bone regeneration for orthopedic implant materials. The scoring system has seven parameters including amount of new bone formation, quality of new bone, bridging, tetracycline‐calcein double labeling, neo‐vascularization, number of inflammatory cells, and amount of fibrous tissue. A minimum of score “0” and maximum of “4” were set for each parameter. For example, higher amounts of newly formed bone with near normal appearance in the extraction socket result in a highest score (score 4) in the “amount of new bone formation” parameter. Similarly for tissues response to the implant material, packed with inflammatory cells and extensive band of fibrous tissues end up in a highest score (score 4) in the “Number of Inflammatory Cells” and “Amount of Fibrosis Tissue” parameters, respectively.

**TABLE 1:** Histopathology scoring parameters (with modifications based on ISO 10993‐6, 2016)

Parameter	Score				
	0	1	2	3	4
Amount of new bone formation	No bone ingrowth	Little amount of bone formation	Moderate amount of bone formation	Large amount of bone formation with voids	Near normal appearance
Quality of new bone	No bone or osteogenic islands	Osteoid, osteogenic islands	Mostly woven bone	Mixture of woven and lamellar bone	Mostly lamellar bone
Bridging	None	Little stumps	Bridging with large gaps	Bridging with little gaps	Bridged
Tetracycline‐Calcein double Labeling	None	Little amount	Moderate amount	Large amount	Near normal
Neo‐vascularization	Absent	Minimal capillary proliferation, focal, 1–3 buds	Group of 4–7 capillaries with supporting fibroblastic structures	Broad band of capillaries with supporting fibroblastic structures	Extensive band of capillaries with supporting fibroblastic structures
Number of inflammatory cells^a^	Absent	Rare	Moderate infiltrates	Heavy infiltrates	Packed
Amount of fibrous tissue	Absent	Narrow band	Moderately thick band	Thick band	Extensive band

^a^ Inflammatory cells include polymorphonuclear cells, lymphocytes, plasma cells, macrophages, mast cells, and multinucleated giant cells.

Histopathological scoring was performed on two slides from each extraction site (P2, P3, and P4) using Masson's trichrome stained sections. Additionally, two slides from each extraction site were left unstained for fluoroscopy. Histopathological slides were meticulously examined and graded blindly by a pathologist, Dr Mei‐Shu Shih specializing in bone and orthopaedics, using the histopathology score listed in Table 1.

### 2.8 Statistical analysis

A non‐parametric Mann–Whitney test with the Bonferroni correction were used to compare the difference between groups. All data were presented as means ± standard error (SE). Statistical analysis was performed by using SPSS software, version 17 (IBM Corp., Armonk, NY). *p* value <0.05 was considered statistically significant. The significance level after Bonferroni correction was set at *α* < 0.017 (*α* = *p*/*k*, where *p* = 0.05, *k* is the number of groups = 3).

## 3 RESULTS

In the post‐operative period, the dogs did not show any complications. The postoperative wound healing around the surgical sites in all the dogs was normal and no dog needed any intensive care. The specimen tissues were well preserved at the sacrifice and free from inflammation.

### 3.1 Biomechanical analysis at 24‐week

**FIGURE 1:**
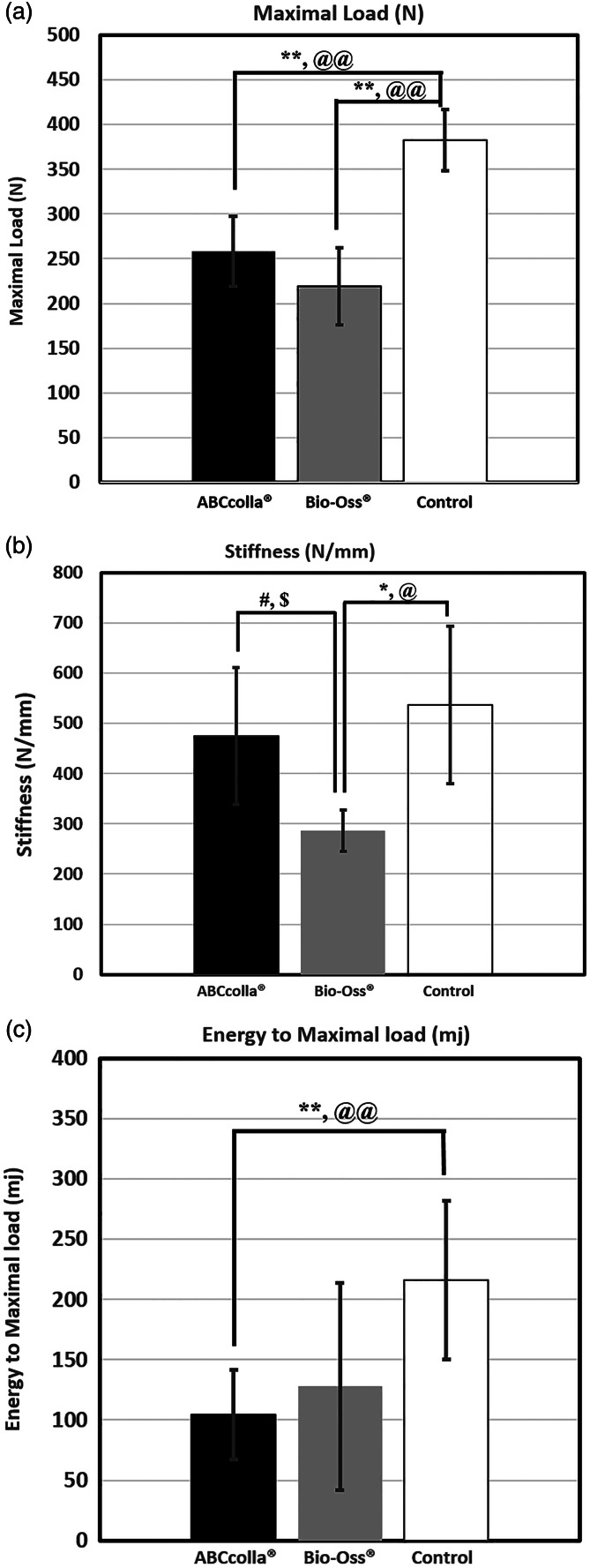
Biomechanical analysis of extraction socket in dog mandible after 24 weeks of healing. The biomechanical parameters included (a) Maximal load, (b) Stiffness, and (c) Energy to maximal load. The black, gray, and white color bars were used to represent ABCcolla®, Bio‐Oss®, and control groups, respectively. Results were expressed as the means ± standard error (*n* = 6). *: *p* < 0.05, **: *p* < 0.01‐ significantly different between ABCcolla® or Bio‐Oss® and unfilled control; #: *p* < 0.05 ‐ significantly different between ABCcolla® and Bio‐Oss®; @: Bonferroni corrected *p* < 0.017, @@: Bonferroni corrected *p* < 0.0033 ‐ significantly different between ABCcolla® or Bio‐Oss® and unfilled control; $: Bonferroni corrected *p* < 0.017 ‐ significantly different between ABCcolla® and Bio‐Oss®

The results of the biomechanical analysis, including maximal load, stiffness, and energy to maximal load, were expressed as the means ± standard error in Figure 1. ABCcolla® treated sites did not show any significant (*p* > 0.05) differences compared to Bio‐Oss® treated defects in maximal load and the energy to maximal load. However, the unfilled control sites showed a significant increase in maximal load than those of ABCcolla® and Bio‐Oss® treated defects (Figure 1(a); *p* < 0.01) and significant greater in the energy to maximal load than that of ABCcolla® (Figure 1(c); *p* < 0.01). In addition, the stiffness of the Bio‐Oss® treated defects were significantly decreased compared to the ABCcolla® treated defects and control; whereas, no significant difference was found between ABCcolla® treated defects and unfilled control sites (Figure 1(b)). The significant differences between groups in maximal load, stiffness, and energy to maximal load mentioned above were not changed even after a Bonferroni correction. Our results demonstrated that ABCcolla® and Bio‐Oss® treated defects showed similar characteristics in both maximal load and energy to maximal load; however, ABCcolla® treated defects exhibited higher stiffness than that of Bio‐Oss® treated sites.

### 3.2 Microradiographs of the mandible

**FIGURE 2:**
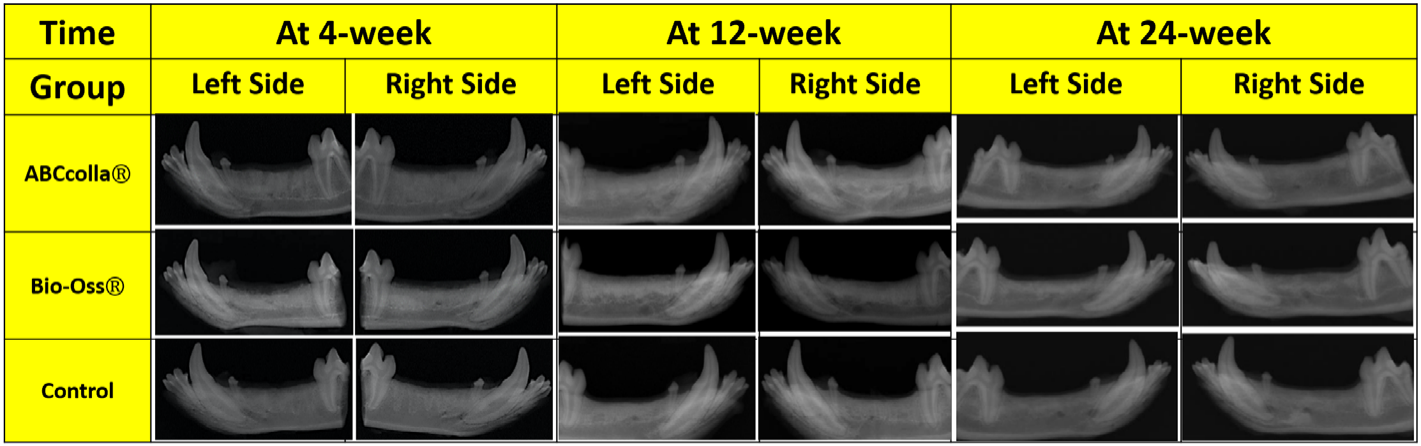
Microradiographs of the mandible taken at 4‐, 12‐, and 24‐week after tooth extraction. Representative X‐ray photographs of SCCO_2_ decellularized bone graft on bone regeneration in a canine extraction socket model at 4‐week, 12‐week, and 24‐week after healing

Microradiographs of the mandible were taken at 4‐, 12‐, and 24‐week after tooth extraction and are shown in Figure 2. The heterogeneity of the degree of mineralization was evident in all healed extraction sockets including the unfilled control sites. Radiopacity was evaluated between groups based on the comparative visual expertise of the veterinarian who performed the microradiographs. Microradiographs in the ABCcolla® treated defects revealed strong radiopacity at 24‐week compared to the Bio‐Oss® group and the unfilled control group (Figure 2(c)).

### 3.3 Histopathology analysis

The mean values of histopathological score in each parameter and their statistical comparison were shown in Table 2. Among the seven parameters evaluated, there was no statistically significant difference between the groups studied at any time point in terms of quality of new bone, neo‐vascularization, and number of inflammatory cells. The ABCcolla® treated defect at 24‐week after socket healing showed a significant higher score (*p* < 0.05) than that of Bio‐Oss® in new bone formation, and demonstrated no difference with that of control untreated site. In the evaluation of bridging, a significant higher score was observed in ABCcolla® treated defect at 24‐week after socket healing, while Bio‐Oss® presented the lowest score (*p* < 0.01). However, both ABCcolla® and Bio‐Oss® treated defects showed a significant higher score (*p* < 0.01) in bridging than that of control at 12‐week. A significant higher amount of tetracycline‐calcein double labeling was identified in the Bio‐Oss® group (*p* < 0.01) at 24‐week. In the evaluation of amount of fibrous tissue, a significant higher score was noticed in the ABCcolla® treated defects at 4‐week, indicating more fibrous tissue present in the socket during early healing. The significant differences between groups in the amount of new bone formation, bridging, tetracycline‐calcein double labeling, and the amount of fibrous tissue mentioned above were remain significant even after a Bonferroni correction. In summary, at 24‐week after socket healing ABCcolla® treated defects demonstrated significantly more new bone formation and better bone bridging, but less amount of fluorescent labeling than those of the Bio‐Oss® group.

**TABLE 2:** Histopathological findings during the healing of the extraction socket at various post‐operative intervals

Groups	Time (weeks)	Amount of new bone formation	Quality of new bone	Bridging	Tetracycline‐Calcein double Labeling	Neo‐vascularization	Number of inflammatory cells	Amount of fibrous tissue
ABCcolla®	4	3.3 ± 0.33	1.8 ± 0.17	3.7 ± 0.33	3.5 ± 0.34	3.3 ± 0.33	1.8 ± 0.31	2.2 ± 0.17^#,$^
Bio‐Oss®	4	2.8 ± 0.48	1.7 ± 0.21	2.8 ± 0.48	2.7 ± 0.42	3.0 ± 0.37	1.7 ± 0.33	2.0 ± 0.45
Control	4	2.8 ± 0.40	1.8 ± 0.17	2.8 ± 0.40	2.8 ± 0.40	2.7 ± 0.33	1.3 ± 0.21	1.3 ± 0.21^#,$^
ABCcolla®	12	4.0 ± 0.0	2.3 ± 0.21	4.0 ± 0.0^##,$$^	1.3 ± 0.21	4.0 ± 0.0	1.2 ± 0.31	1.2 ± 0.31
Bio‐Oss®	12	3.8 ± 0.17	2.5 ± 0.22	4.0 ± 0.0^##,$$^	2.5 ± 0.43	4.0 ± 0.0	1.5 ± 0.22	1.7 ± 0.21
Control	12	3.3 ± 0.33	2.2 ± 0.17	1.0 ± 0.0^##,$$^	2.0 ± 0.37	4.0 ± 0.0	1.3 ± 0.33	1.7 ± 0.21
ABCcolla®	24	3.7 ± 0.21^*,@^	3.0 ± 0.26	3.7 ± 0.21^**,@@,#,$^	2.0 ± 0.37^**,@@^	3.0 ± 0.26	1.5 ± 0.22	1.5 ± 0.22
Bio‐Oss®	24	2.2 ± 0.31^*,@^	2.8 ± 0.17	1.7 ± 0.33^**,@@,#,$^	4.0 ± 0.00^**,@@,##,$$^	2.8 ± 0.48	2.8 ± 0.48	2.0 ± 0.26
Control	24	3.3 ± 0.33	3.0 ± 0.0	2.8 ± 0.17^#,$^	2.3 ± 0.21^##,$$^	3.3 ± 0.21	2.5 ± 0.34	2.0 ± 0.26

*Note*: Results were expressed as the means ± SE. Significantly different between ABCcolla® or Bio‐Oss® and Control ‐ #: *p* < 0.05; ##: *p* < 0.01. Significantly different between ABCcolla® and Bio‐Oss® ‐ *: *p* < 0.05; **: *p* < 0.01. Significantly different between ABCcolla® or Bio‐Oss® and Control ‐ $: Bonferroni corrected *p* < 0.017; $$: Bonferroni corrected *p* < 0.0033. Significantly different between ABCcolla® and Bio‐Oss® ‐ @: Bonferroni corrected *p* < 0.017; @@: Bonferroni corrected *p* < 0.0033.

**FIGURE 3:**
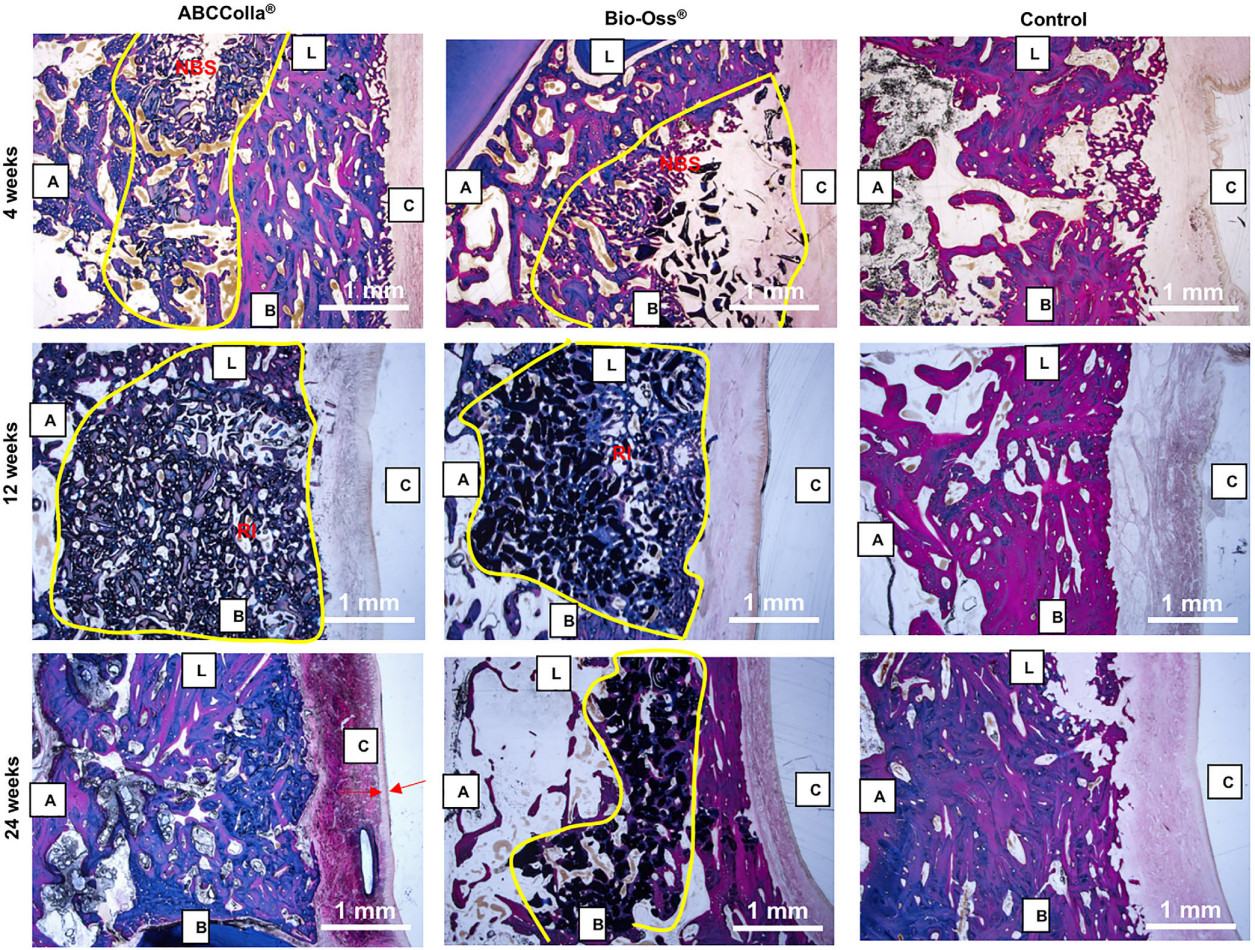
Representative histomorphometric photographs of Masson's trichrome staining of the extraction sockets after implantation of ABCcolla® (left column), Bio‐Oss® (middle column), or unfilled control (right column) at 4‐ (upper row), 12‐ (middle row), and 24‐week (bottom row) of healing. The exact area of the samples being depicted has been marked with solid yellow line. Scale bars are 1.0 mm. B: buccal; L: lingual; C: coronal; A: apical. NBS‐new bone spicules; RI‐remnant of implant material; Arrows indicates membrane

**FIGURE 4:**
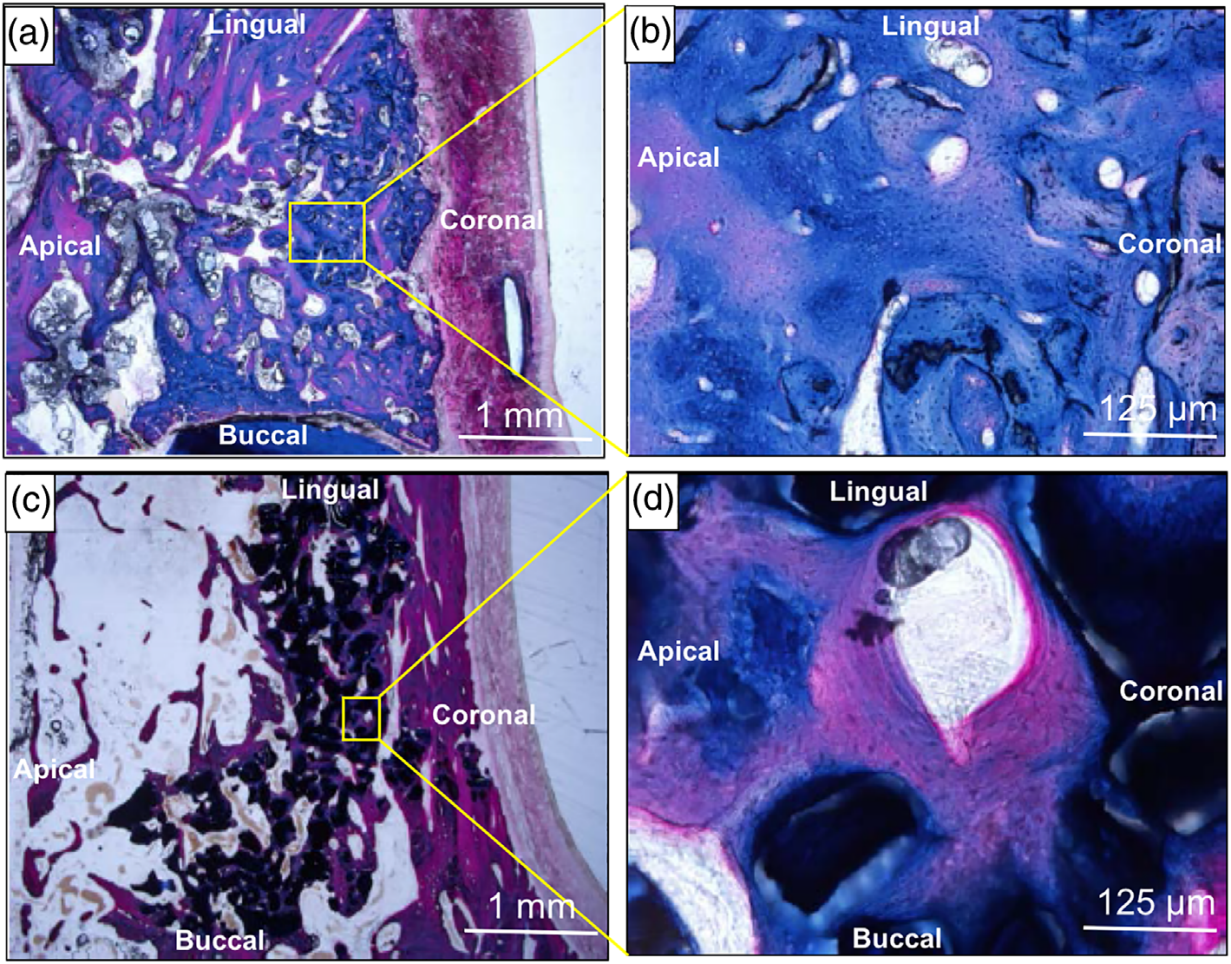
Representative photomicrographs of Masson's trichrome staining of the extraction sockets after implantation of either ABCcolla® or Bio‐Oss® at 24‐week after socket healing. (a) ABCcolla®; (b) 8X higher magnification of yellow square in (a); (c) Bio‐Oss®; and (d) 8X higher magnification of yellow square in (c). Scale bars are 1.0 mm in (a) and (c); whereas, 125 μm in (b) and (d)

**FIGURE 5:**
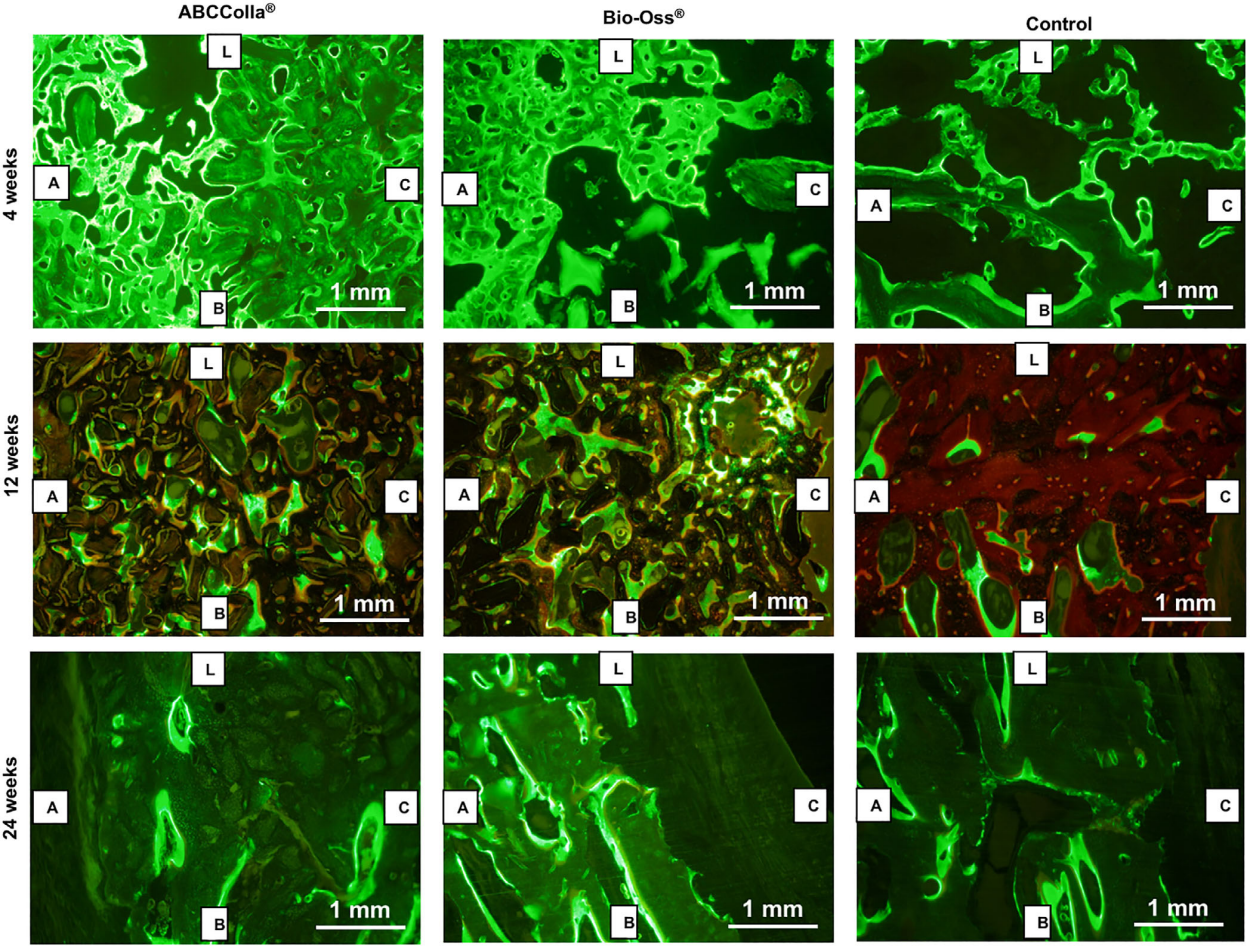
Representative fluorescent micrographs of extraction socket at 4‐ (upper row), 12‐ (middle row), and 24‐week (bottom row) after healing by ABCcolla® (left column), Bio‐Oss® (middle column), or unfilled treatment (right column). Fluorescent intensity indicates bone mineralization. Scale bars are 1.0 mm. B: buccal; L: lingual; C: coronal; A: apical

The microscopic examination demonstrated the healing, repair and regeneration of ABCcolla® were comparable to those of the Bio‐Oss® group. The Masson's trichrome staining (Figure 3) demonstrated no significant alterations in the histological features between the ABCcolla® and Bio‐Oss® groups at both 4‐ and 12‐week. These two treatment groups all showed similar amounts of new bone spicules at 4‐week and remnant of implant material at 12‐week. In fact, there were no statistically significant difference in the amounts of residual grafting material between ABCcolla® and Bio‐Oss® group (Appendix S1). Inflammation and fibrosis were low to moderate, which could be the results of constant chewing pressure on the newly formed bone and the remnant implant particulate material. A relatively large portion of newly formed bone was observed in the ABCcolla® group compared to the Bio‐Oss® group at 24‐week (Figure 4). Fluorescent micrographs of the healed socket (Figure 5) revealed larger amounts of bright fluorescence at 4‐week in the ABCcolla® group compared to both the Bio‐Oss® and control groups, indicating active mineralization. However, the amounts of bright florescence were subsequently decreased at 12‐week and 24‐week in the ABCcolla® group. Interestingly, the relative florescence was higher in the Bio‐Oss® group compared to the ABCcolla® and control groups at 24 week, indicating mineralization was complete.

## 4 DISCUSSIONS

Bone substitutes, such as porcine bone xenografts, have been demonstrated to be an excellent grafting material for bone augmentation procedures. Clinical studies using cortical porcine bone showed excellent results and promising clinical application (Scarano et al., 2011). Previously, we have demonstrated that the regenerative ability of porcine grafting material can be improved by a decellularization process using SCCO_2_ extraction technology (Huang et al., 2017). By employing this novel SCCO_2_ extraction technology, the cellular components of porcine corneas were efficiently removed, and the biomechanical properties of the scaffold were well preserved. In addition, the acellular corneas exhibited no immunological reactions, displayed good biocompatibility and long‐term stability. We concluded that acellular porcine cornea developed by SCCO_2_ extraction technology was a suitable scaffold for corneal wound healing (Huang et al., 2017). In the present study, ABCcolla® was achieved by using this novel SCCO_2_ extraction technology for bone regeneration. Validation of the decellularization process of ABCcolla® was performed in our laboratory by hematoxylin and eosin staining and DNA analysis (manuscript submitted to Journal of Tissue Engineering and Regenerative Medicine for publication and is currently under revision). After decellularization, the chemical contents and structure of ABCcolla® were found to be similar to those of human grafts. Furthermore, in vitro and in vivo biocompatibility of ABCcolla® in rabbits revealed no significant differences compared to Bio‐Oss® in bone formation at 4, 12 and 24 week.

The present preclinical study compared two similar inorganic bone grafts from different animal species. It has been reported that the placement of a barrier membrane greatly improved the potential for bone regeneration in a surgically created defect (Nakajima et al., 2007). In a preclinical study, M. Kim, Kim, et al. (2008) evaluated the efficacy of inorganic bone combined with a resorbable collagen membrane in regenerating an alveolar ridge defect. They found a significant increase in bone density when using inorganic bone combined with resorbable collagen membrane evaluated by CT compared to only bone or unfilled control. It has been reported that lack of a barrier membrane may have a negative bone regeneration effect due to lack of graft containment and wound stabilization (D. M. Kim, Hong, et al., 2017). Therefore, the bone regenerative effect of the ABCcolla® was done in combination with a commercial membrane, Bio‐Gide®, in a canine mandible extraction socket. In the present study, a comparative analysis of the ABCcolla® and the Bio‐Oss® bone graft was done to evaluate bone regeneration in a canine tooth extraction socket model.

The results of the present study indicate that new bone formation when using ABCcolla® is promising. However, there are few limitations to this study. First, the Mann–Whitney test (a nonparametric statistical test) was used to identify the difference between the means of two independent groups in this study due to ordinal data within small sample size. It should be noted that tests for the difference between the means of two independent groups in this study can be subject to low power. Second, clustered randomization was selected for this study in which the individual dog, not the extraction sockets, was randomly allocated for different treatments. In other words, the six extraction sockets (P2 to P4, bilateral) in an individual dog were all assigned in one treatment modality. It has been reported that clustered randomization could result in loss of efficiency, particularly in terms of effective sample size (Hauck et al., 1991). Nevertheless, it may also create some issues if all the six extraction sockets were randomly assigned to three different treatment groups. The defect sizes of the P2 to P4 extraction sockets were different and may have an impact on bone regeneration. This is the rationale that we select cluster randomization by allocate treatment to individual dog instead of extraction site. Lastly, caution should be taken when interpreting the mean data in the histopathology scores because the ordinal numbers from 0 to 4 were used in the scoring system. Although, the ordinal method is commonly used by pathologists for the evaluation of a medical device, it does make the data interpretation more challangings (Gibson‐Corley et al., 2013). In the ordinal type of data, samples were allocated to a category exhibiting an ordered progression in degree of severity, such as from score 0 (absent) to 4 (extensive) in this study. The problem of the ordinal data is that they are separated by unequal intervals. For example, a score of 4 is not necessarily 4 times as more as a score of 1. Furthermore, the interval between a score of 1 and 2 is not necessarily the same as the interval between a score of 2 and 3. Therefore, the use of these type of scores in mathematical calculation is considered statistically inappropriate (Green et al., 2014). It has been reported that it is necessary for the histopathological slides examined and graded blindly by a pathologist to minimize observational bias (Gibson‐Corley et al., 2013). It is also recommended that 4–5 score levels may be an ideal range to increase detection and repeatability in a scoring system (Shackelford et al., 2002). In order to frequently produce meaningful and valid scoring data in the histopathological analysis, we have followed the key principals as mentioned above for the development of semiquantitative scoring system via histologic examination in this study. Moreover, a Bonferroni correction was applied to recheck the status of significant difference since this study contained three comparison groups. The significant differences were not altered even after a Bonferroni Correction.

## 5 CONCLUSIONS

Within the limits of the study, we concluded that ABCcolla® treated defects demonstrated significantly more new bone formation and better bone bridging, but less amount of fluorescent labeling than those of the Bio‐Oss® group. However, clinical studies in humans are recommended to confirm these findings.

## CONFLICT OF INTEREST

The authors declare that they have no competing interests.

## Supporting information

**Appendix S1.** Supporting information.

## Data Availability

The data that supports the findings of this study are available in the supplementary material of this article and they are also available from the corresponding author upon request.
