# Supplementary material for: Evaluating the bone‐regenerative role of the decellularized porcine bone xenograft in a canine extraction socket model
Source: Clin Exp Dent Res. 2020 Dec 1;7(4):409–18. doi: 10.1002/cre2.361 (PMC8404509; doi:10.1002/cre2.361)
Supplement: Supplementary file 1 — Appendix S1. Supporting information. [file CRE2-7-409-s001.zip › CRE2_361_cre2.20200117-File009.docx]

Supplementary Table: Residual grafting material in the extraction socket at various post-operative intervals.

| Groups | Time (weeks) | Residual Grafting Material |
| --- | --- | --- |
| ABCcolla® | 4 | 2.2±0.31 |
| Bio-Oss® | 4 | 1.8±0.31 |
| Control | 4 | 0 |
| ABCcolla® | 12 | 2.5±0.67 |
| Bio-Oss® | 12 | 3.8±0.17 |
| Control | 12 | 0 |
| ABCcolla® | 24 | 2.2±0.17 |
| Bio-Oss® | 24 | 2.0±0.37 |
| Control | 24 | 0 |

Results were expressed as the means ± SE.
