# Supplementary figures and images for: Evaluating the bone‐regenerative role of the decellularized porcine bone xenograft in a canine extraction socket model
Source: Clin Exp Dent Res. 2020 Dec 1;7(4):409–18. doi: 10.1002/cre2.361 (PMC8404509; doi:10.1002/cre2.361)

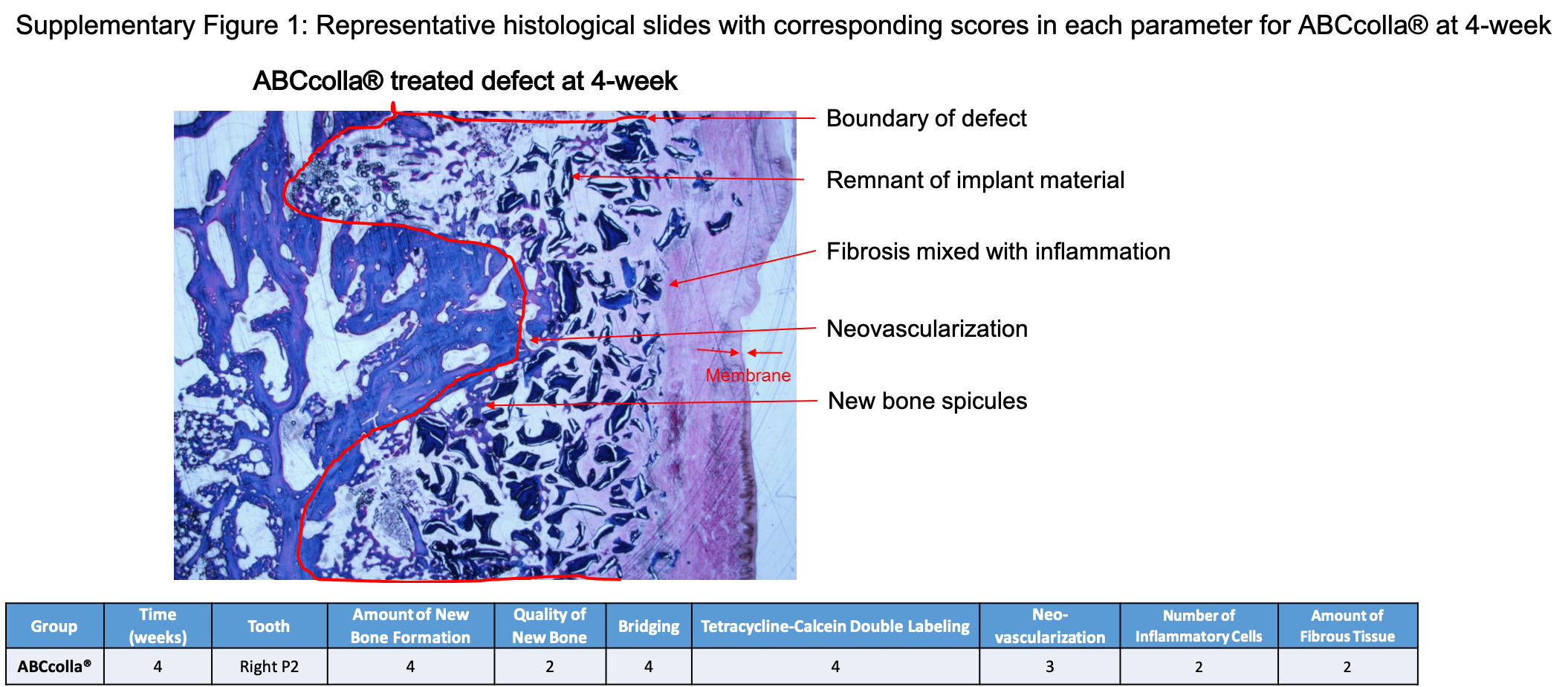

Supplement: Supplementary file 1 — Appendix S1. Supporting information. [file CRE2-7-409-s001.zip › CRE2_361_cre2.20200117-File010.png]

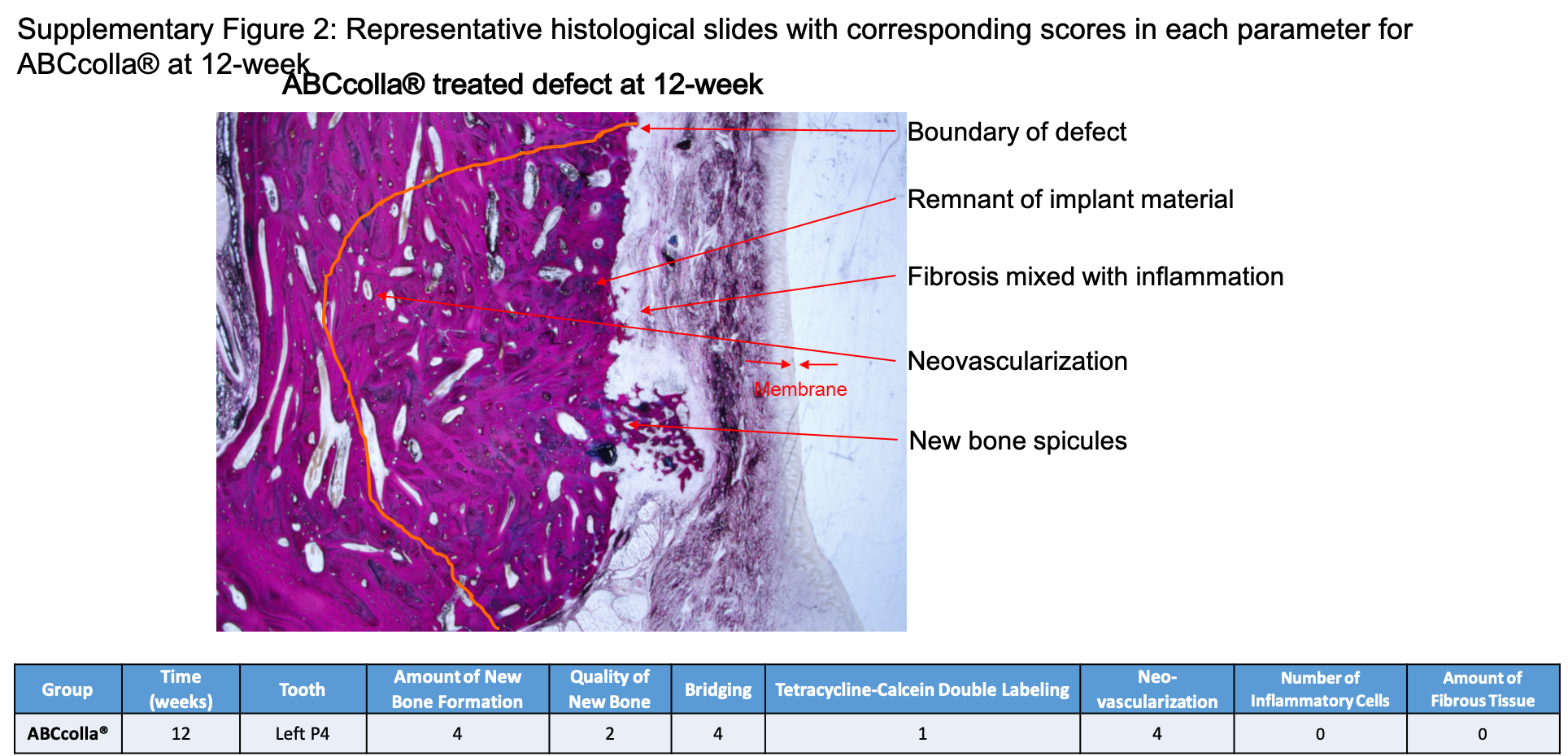

Supplement: Supplementary file 1 — Appendix S1. Supporting information. [file CRE2-7-409-s001.zip › CRE2_361_cre2.20200117-File011.png]

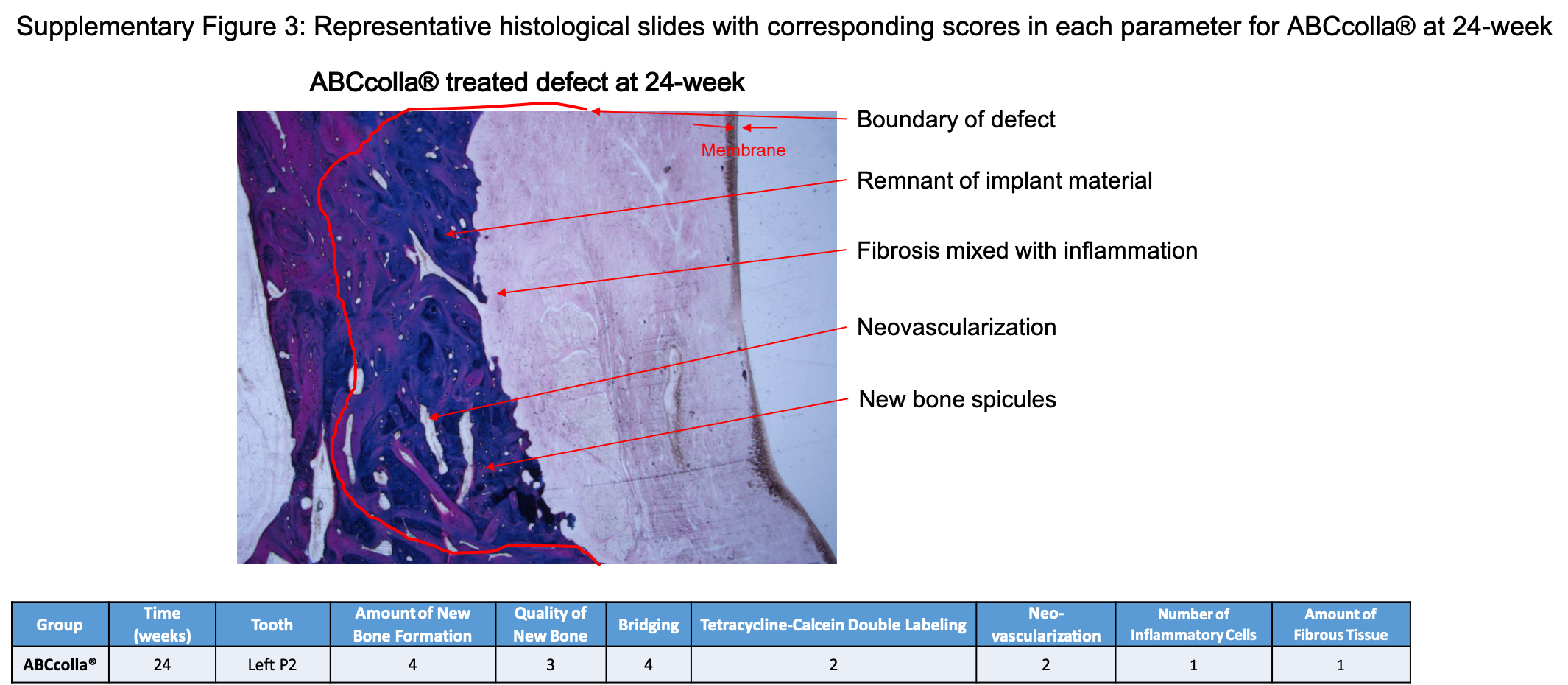

Supplement: Supplementary file 1 — Appendix S1. Supporting information. [file CRE2-7-409-s001.zip › CRE2_361_cre2.20200117-File012.png]

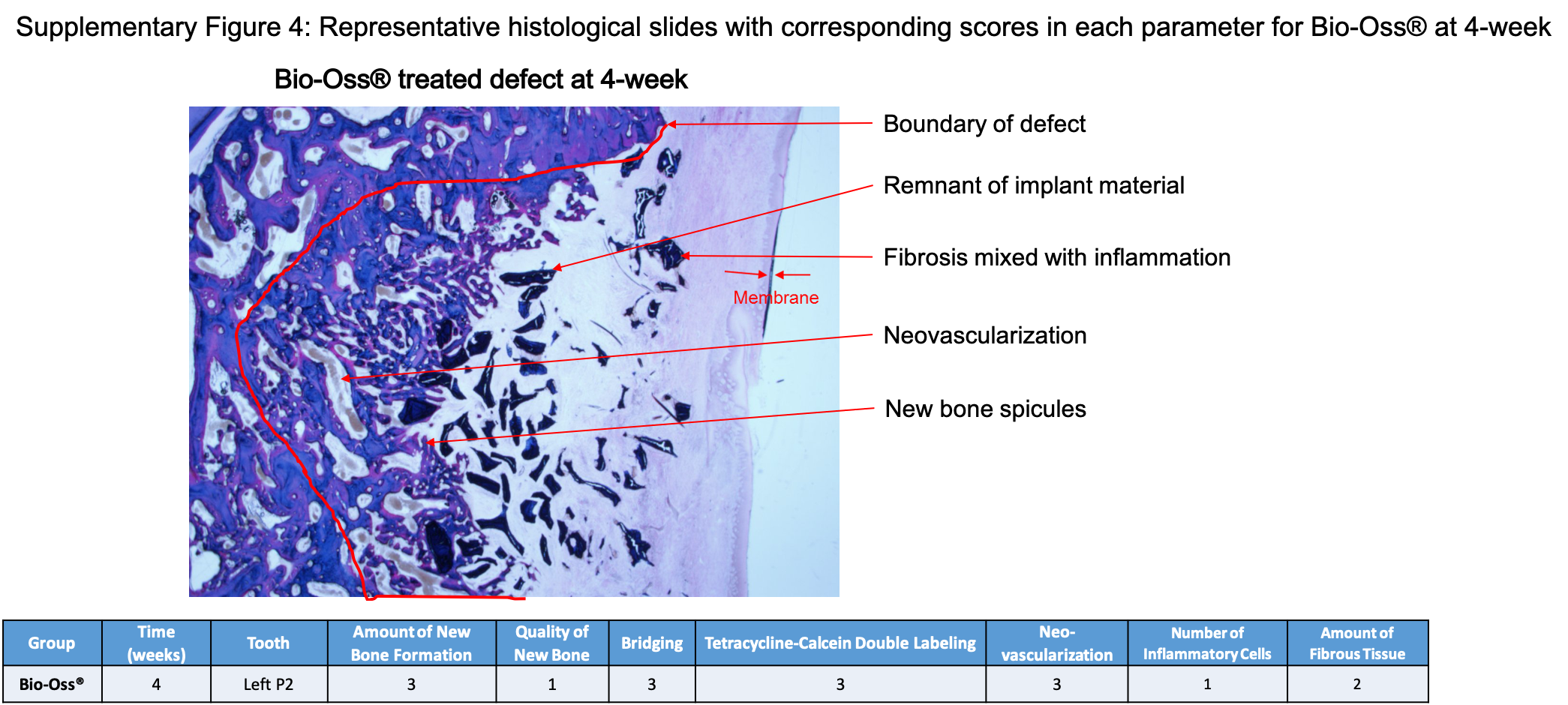

Supplement: Supplementary file 1 — Appendix S1. Supporting information. [file CRE2-7-409-s001.zip › CRE2_361_cre2.20200117-File013.png]

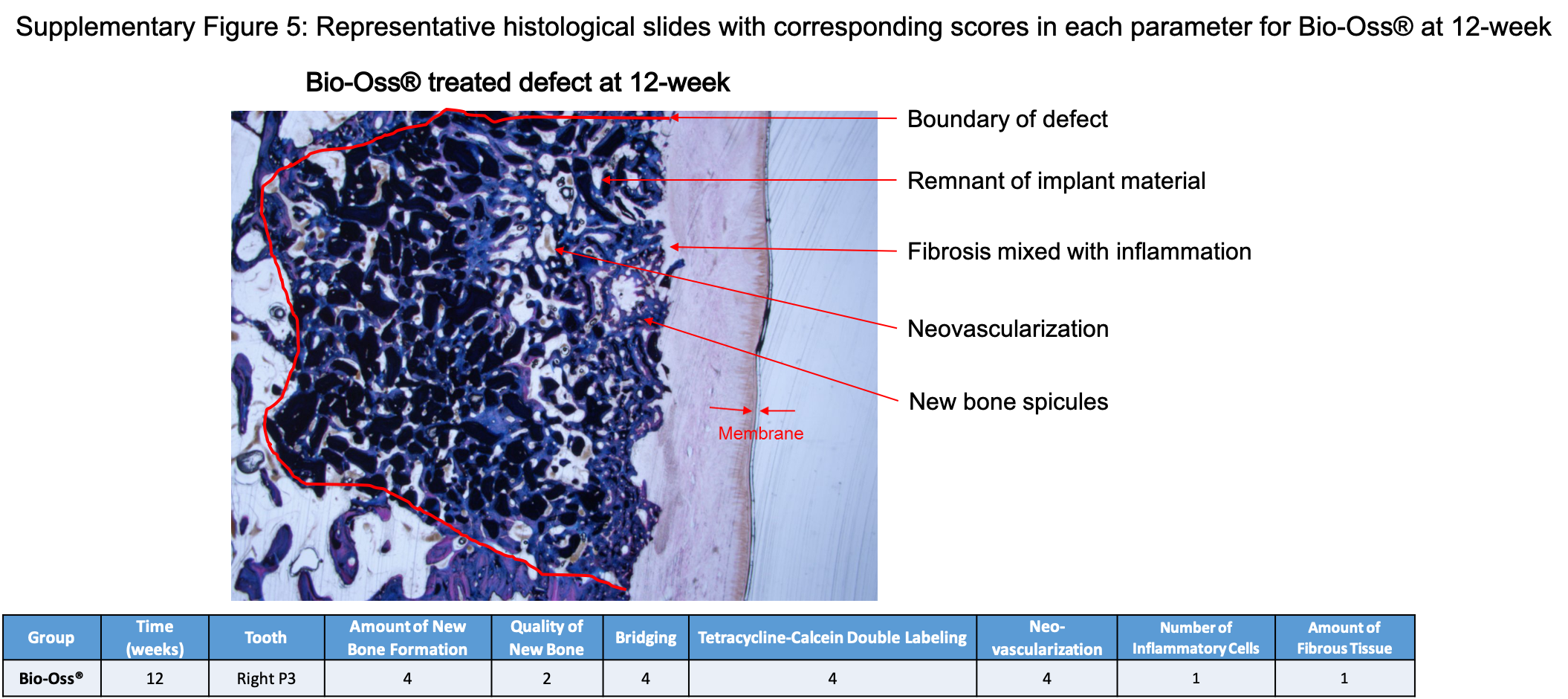

Supplement: Supplementary file 1 — Appendix S1. Supporting information. [file CRE2-7-409-s001.zip › CRE2_361_cre2.20200117-File014.png]

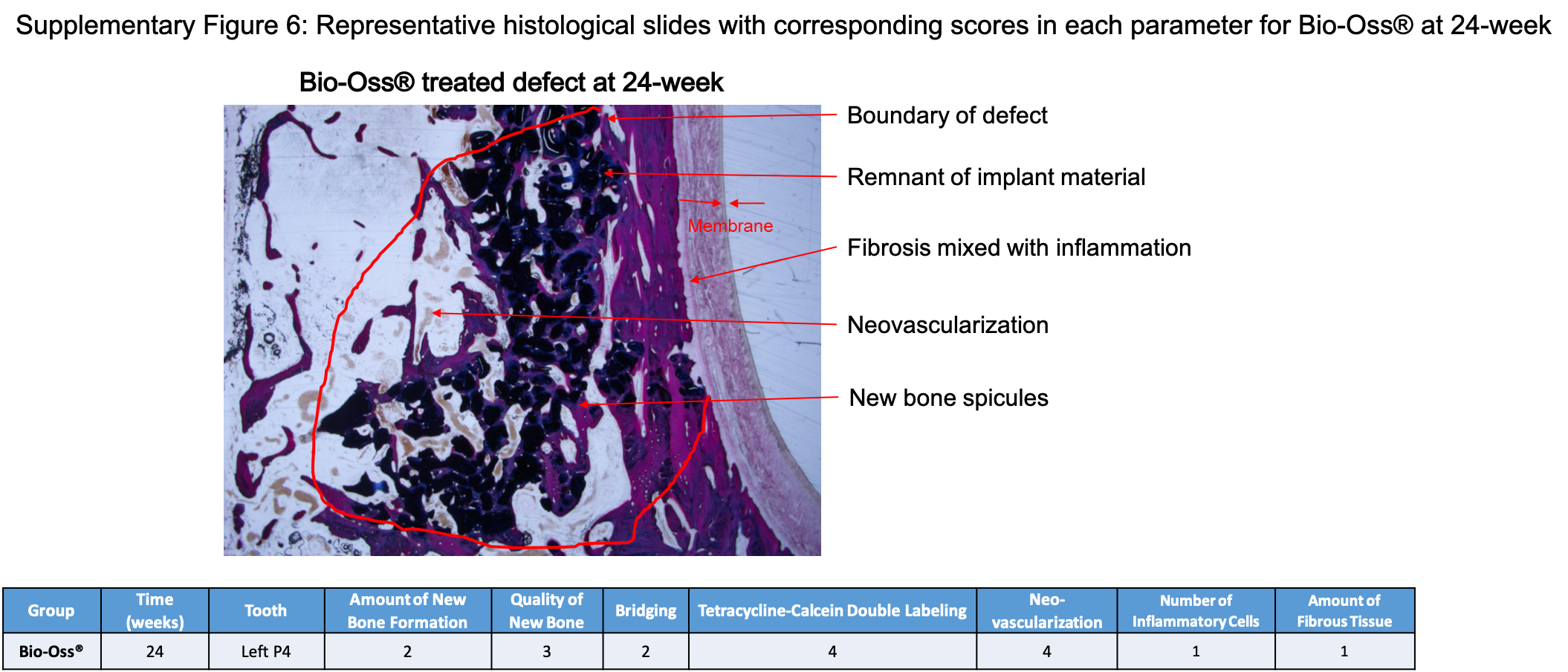

Supplement: Supplementary file 1 — Appendix S1. Supporting information. [file CRE2-7-409-s001.zip › CRE2_361_cre2.20200117-File015.png]
